# Do Sex Ratio Distorting Microbes Inhibit the Evolution of Pesticide Resistance? An Experimental Test

**DOI:** 10.1111/eva.70003

**Published:** 2024-10-14

**Authors:** Adam M. Fisher, Amelia‐Rose V. McKenzie, Tom A. R. Price, Michael B. Bonsall, Robert J. Knell

**Affiliations:** ^1^ School of Biological and Behavioural Sciences Queen Mary University of London London UK; ^2^ School of Natural Sciences University of Hull Hull UK; ^3^ Department of Evolution, Ecology and Behaviour University of Liverpool Liverpool UK; ^4^ Department of Zoology University of Oxford Oxford UK

**Keywords:** adaptation, biocide, insecticide, pesticide, resistance, sex ratio, skew, *Spiroplasma*

## Abstract

We are still largely reliant on pesticides for the suppression of arthropod pests which threaten human health and food production, but the recent rise of evolved resistance among important pest species has reduced pesticide efficacy. Despite this, our understanding of strategies that effectively limit the evolution of resistance remains weak. Male‐killing sex ratio distorting microbes (SRDMs), such as *Wolbachia* and *Spiroplasma*, are common among arthropod species. Previous theoretical work has suggested that they could limit adaptive potential in two ways: first, because by distorting sex ratios they reduce the effective population size, and second, because infected females produce no male offspring which restricts gene flow. Here we present the results of a novel experiment in which we test the extent by which these two mechanisms limit the adaptive response of arthropods to pesticide. Using a fully factorial design, we manipulated the adult sex ratio of laboratory populations of *Drosophila melanogaster*, both in the presence and absence of SRDMs, and exposed these populations to six generations of pesticide poisoning. This design allows the effects of SRDMs on sex ratio and their effects on gene flow to be estimated separately. After six generations, individuals from populations with even sex ratios displayed a higher resistance to pesticide relative to individuals from female‐biased populations. By contrast, we found no effect of the presence of SRDMs in host populations on pesticide resistance independent of sex ratio. In addition, males were more susceptible to pesticide than females—this was true of flies from both naïve and previously exposed populations. These findings provide the first empirical proof of concept that sex ratio distortion arising from SRDMs can limit adaptation to pesticides, but cast doubt on the theoretical effect of male‐killers limiting adaptation by disrupting gene flow.

## 1 Introduction

Arthropod pests impose a heavy and persistent burden on food production and human health, and the successful suppression of pest populations increases food security and saves lives. As an example, the use of insecticide‐treated bed nets and insecticide sprays is estimated to have prevented half a billion malaria‐related deaths between 2000 and 2015 (Bhatt et al. 2015). Due to long‐term and widespread pesticide use, however, the frequency of pesticide resistance in arthropods has been increasing over time, rendering many pesticides far less effective than they once were (Gould, Brown, and Kuzma 2018; Tabashnik, Brévault, and Carrière 2013; Whalon, Mota‐Sanchez, and Hollingworth 2008). Moreover, the warming climate is expected to exacerbate this rise in pesticide resistance by boosting pest reproductive rates (Ma et al. 2021; Maino, Umina, and Hoffmann 2018). Technologies and strategies that aim to suppress pests without the use of pesticides are being developed, but many of these solutions are either species‐specific, or are not yet ready for use (Athanassiou et al. 2018; Legros et al. 2021). For the time being, therefore, we are still largely reliant on conventional pesticides, and must seek ways to enhance their long‐term effectiveness by minimising pesticide resistance.

In recent years, theoretical models have shown that the efficacy of pesticides can be bolstered by combining pesticide treatments with the manipulation of pest reproductive biology. A mathematical modelling study based on the lifecycle of the gypsy moth, *Lymantria dispar*, showed that the disruption of mating dynamics using false pheromones lowers the time taken for pesticides to drive pests extinct (Blackwood et al. 2012). Similarly, the release of sterile males into pest populations lowers pest reproductive rates, making pests more vulnerable to rapid decline upon pesticide exposure (Lampert and Liebhold 2021). In theory, these strategies can reduce the number of generations needed for pesticides to eradicate pest populations, reducing the likelihood that pesticide resistance will emerge. Unfortunately, these strategies also require persistent human intervention, meaning their ecological effectiveness and cost viability diminishes with time and pest density (Lampert and Liebhold 2021). As such, their potential use is likely mostly to be limited to emerging rather than established pest populations. In addition, our understanding of how to limit arthropod adaptation to pesticides largely rests on theoretical findings, and empirical support for most of these strategies is lacking.

Here, we examine the potential of maternally inherited sex ratio distorting microbes (SRDMs) to alter the rate of adaptation of arthropods to pesticide exposure. SRDMs are common among arthropod species (Weinert et al. 2015) and because they can only be transmitted by females, many of them have evolved to disrupt the reproductive ecology of host populations by biasing the sex ratios of host broods towards females. This is typically achieved by killing the male offspring of hosts, feminising male offspring, or inducing parthenogenesis (Engelstädter and Hurst 2009). As a result, infected populations often exhibit heavily female‐biased sex ratios. A previous theoretical study explored the potential utilisation of SRDMs for pest control, and showed that SRDMs can help drive pest populations extinct by inducing mate‐finding Allee effects (i.e., negative density‐dependence) (Berec, Maxin, and Bernhauerová 2016). This result is corroborated by other studies which also show the potential for SRDMs to induce Allee effects (Blackwood, Vargas Jr, and Fauvergue 2018; Hatcher et al. 1999). However, these studies also find that a vertical SRDM transmission rate of ⪆95% is required to drive populations extinct. Given that SRDM transmission is known to be temperature sensitive (Corbin et al. 2021; Thomas and Blanford 2003), such high transmission rates are unlikely to persist in the field (Fisher et al. 2022), undermining the practicality of SRDMs as agents of pest control. However, there is another currently unexplored utilisation of SRDMs for pest control, and that is the potential for SRDMs to slow the long‐term evolutionary response to pesticides.

There are two main reasons to predict that SRDMs can limit the evolution of pesticide resistance in host populations. First, under random mating conditions, effective population size (*𝑁*_𝑒_) is predicted to decline as the operational sex ratio (OSR—the sex ratio of reproductive individuals) deviates from even due to there simply being fewer genetically unique reproductive pairings in the population (Charlesworth 2002; Chiba et al. 2023; Wright 1931). Second, because infected females produce female‐only broods, alleles that incur resistance that are carried by infected individuals will be confined to the infected portion of population; thus limiting gene flow of beneficial alleles through the population as a whole (Engelstädter and Hurst 2007). Similarly, resistance alleles carried by males that mate only with infected females will also become restricted to the infected portion of the population, as their offspring will be infected via the mother. Despite these theoretical predictions, however, to date there have been no empirical studies addressing the use of SRDMs in limiting pesticide resistance, and more generally, empirical data related to the utility of reproductive disruption in limiting pesticide resistance are non‐existent.

In this study, we provide the first (to the best of our knowledge) empirical test of the two mechanisms by which SRDMs might limit adaptation to pesticide exposure. We used a fully factorial experiment in which laboratory populations of *Drosophila melanogaster* had either their sex ratio or the presence of an SRDM (*Spiroplasma poulsonii*), or both, manipulated and were exposed to pesticide over several generations. We tested two main predictions: (1) populations with distorted sex ratios would show reduced adaptation to pesticide due to having a smaller *𝑁*_𝑒_, and (2) populations with the SRDM present would show a reduction in adaptation relative to populations without the SRDM because of the effects of the SRDM on gene flow.

## 2 Methods

### 2.1 Stock Populations and Maintenance

*D. melanogaster* is a common model species for evolution experiments owing to its easy husbandry and short generation time. Moreover, data generated from *D*. *melanogaster* are relevant to certain pest scenarios due to its close phylogenetic relationship to the congeneric species *Drosophila suzukii*, a widely distributed fruit pest (Chiu et al. 2013; Ørsted and Ørsted 2019). To ensure sufficient standing genetic variation for adaptation, we generated laboratory stocks of *D. melanogaster* by crossing several hundred individuals from two outbred laboratory populations: Dahomey and Canton‐S. We used flies from these laboratory populations rather than wild caught flies because these populations were collected prior to the widespread global use of pesticide, meaning our experimental flies were unlikely to have experienced any a priori pesticide selection pressure.

To generate our SRDM‐infected populations, we used females from the Canton‐S population infected with a strain of the bacteria *Spiroplasma poulsonii* crossed with males from both the Canton‐S and Dahomey population. Bacteria of the genus *Spiroplasma* are commonly found in wild arthropod populations and distort sex ratios by killing the male offspring of hosts during embryogenesis. The strain of *S*. *poulsonii* used in this study was originally collected in Uganda (Pool, Wong, and Aquadro 2006) and is known to exhibit a high transmission rate, resulting in heavily female‐biased broods (Jones and Hurst 2020). All stock populations were divided into four sub‐populations comprised of hundreds of individuals contained within large (250 mL) glass bottles. Initially, PCR was used to confirm *S*. *poulsonii* infection in the Canton‐S population with individuals from the uninfected stocks being used as negative controls (see Jones and Hurst 2020 for a detailed PCR protocol); thereafter, we monitored the persistence of *S*. *poulsonii* by examining the sex ratio of a sample of ≈100 individuals from each sub‐population every generation. Sub‐populations in which > 90% of sampled individuals were female were assumed to be infected and were used to seed the next generation of infected flies. Sub‐populations in which the sampled individuals had fewer than 90% females were inspected further by isolating all females of that sub‐population before inspecting the sex ratio of their specific broods; broods in which ≥ 90% of individuals were female were introduced back into the infected population, others were discarded. All flies were kept at 25°C and reared on maize‐sugar‐yeast agar medium.

### 2.2 Pesticide Bioassays

We generated pesticide dose–response curves for all three of the *D*. *melanogaster* phenotypes used in our study (male, uninfected female and infected female). Permethrin was our pesticide of choice because it is used in practice for the suppression of insect disease vectors (especially *Anopheles*), but is vulnerable to a loss of potency following the evolution of resistance (Vulule et al. 1994). To expose individuals, we dissolved a known quantity of permethrin (trans‐cis mixed isomer Scientific Laboratory Supplies Ltd.) in acetone and carried out a serial dilution to give a range of permethrin concentrations from 0 to 1200 μg/mL (specifically: 0, 50, 75, 100, 150, 200, 300, 400, 600, 800 and 1200 μg/mL). We then evenly applied 0.5 mL of the acetone‐permethrin solution to the inside of standard 25×90 mm fly vials. The vials were left for 15 min to allow the acetone to evaporate, leaving behind the permethrin precipitate. Individuals were then separated into phenotype‐specific treated vials (10–20 individuals per vial) where they remained for 1 h. We then removed the individuals and placed them into clean vials for 24 h before recording how many individuals had died. Dose–response bioassays were conducted on both naïve flies (*n*_males_ = 66 vials, *n*_females_ = 67 vials), and flies that had undergone six generations of selection for resistance to permethrin (*n*_males_ = 119 vials, *n*_females_ = 119 vials, see details below). Vials were distributed evenly among the aforementioned concentrations.

### 2.3 Experimental Evolution

**FIGURE 1:**
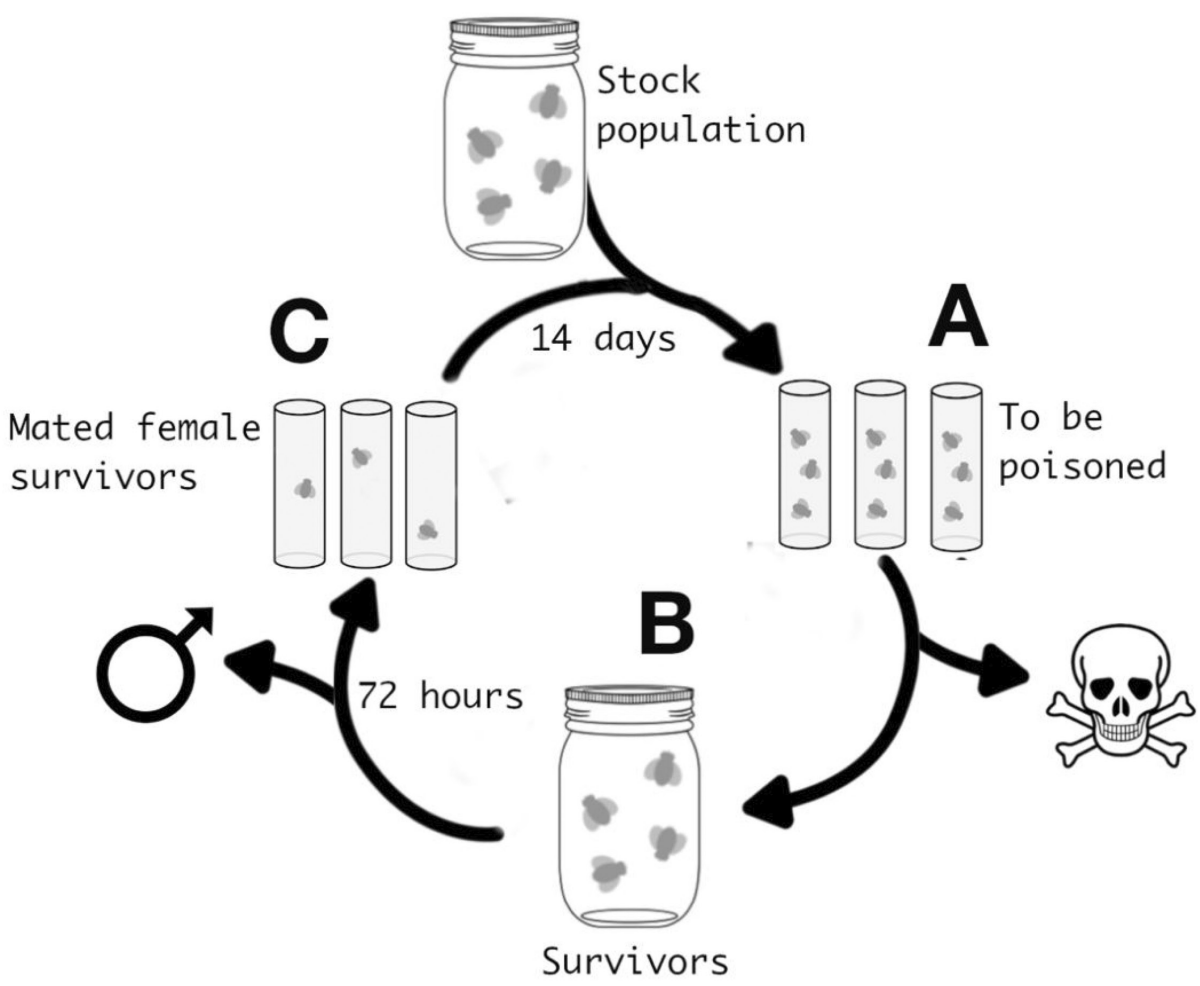
A generational cycle of our experimental evolution (EE) lines using *Drosophila melanogaster*. EE lines were seeded from the stock population before undergoing six generations of permethrin exposure. Labels A, B and C are used to cross‐reference passages of the main text with specific experimental stages.

We exposed experimental evolution (EE) lines of *D*. *melanogaster* to permethrin for six generations and monitored fly mortality over time (Figure 1). Because the effects of SRDMs on adaptation are predicted to occur through two separate mechanisms: (1) the change in *N*e caused by the change in sex ratio, and (2) the restriction on gene flow caused by the presence of the SRDM (Engelstädter and Hurst 2007), we manipulated sex ratio and *S*. *poulsonii* prevalence separately to give a 2 × 2 factorial design with four treatment groups, allowing us to test for an effect of each mechanism independently. The treatment groups were: (1) uninfected population with an even sex ratio, (2) uninfected population with a female biased (3:1) sex ratio, (3) infected population with an even sex ratio and (4) infected population with a female biased (3:1) sex ratio.

For each EE line, the experiment was started using 200 flies from either the infected or the uninfected stock populations, with either 100 males and 100 females (even sex ratio) or 50 males and 150 females (female‐biased sex ratio). These flies were exposed to permethrin using the method described above at a concentration of 70 μg/mL, which pilot bioassays indicated to be approximately an LD_50_ dose for naïve flies (see point A in Figure 1). Immediately after exposure, all of the flies for each EE line were placed together in a 250‐mL glass bottle with maize‐sugar‐yeast agar medium, and left to mate for 72 h (point B in Figure 1). After 72 h, all surviving flies were sexed and counted. Forty surviving females were put into individual vials containing maize–sugar–yeast agar medium for 2 weeks to lay eggs for the next generation, and males were discarded (Figure 1 point C).

After 2 weeks, when the next generation of adults had emerged, 20 of the vials from the previous step were chosen at random. Individuals from these 20 vials were anaesthetised using CO_2_, mixed together, and 200 flies were collected to the appropriate sex ratio. Twenty vials of the initial 40 were used because some females would fail to reproduce, and with this design we could be sure of starting each generation with flies from the same number of families. For the infected treatments, brood sex ratios were examined (as above) to determine which broods were infected (> 90% females). Infected and uninfected females were then selected to appropriate numbers to maintain *S*. *poulsonii* prevalence at roughly 50% in the infected EE lines. Since this could not ever give us an exact 50% prevalence, we recorded the number of infected families used and included this in the analysis as an explanatory variable.

The flies were then exposed to permethrin as before, allowed to mate and their offspring were used for the next generation, for a total of six generations. Each EE line was kept separate, and no flies were transferred between lines. Four replicate lines were produced for each treatment combination for a total of 16 experimental lines. It is worth highlighting that, although the offspring of the 20 families used to seed the next generation emerged from family‐specific vials, our experiment was designed to decrease the amount of inbreeding. For example, females were housed in a mixed population of survivors for 72 h prior to laying (Figure 1) to encourage mating with non‐kin. Moreover, last male sperm precedence (i.e., the male that last mated with the female prior to laying is likely to father the majority of offspring) is commonly observed in *Drosophila* spp. (Laturney, van Eijk, and Billeter 2018), further decreasing the probability of inbreeding.

### 2.4 Statistical Analyses

First, we tested whether flies that had been exposed to pesticide for six generations were more resistant to pesticide than naïve flies, by analysing our dose–response data using a generalised linear model (GLM) with binomial error. Our maximal model included mortality (the number of dead individuals per vial) as the response variable, and the predictor variables were: log permethrin concentration, experimental evolution (EE) line, sex, exposure status (i.e., naïve to pesticide or not), the interaction between concentration and sex and the interaction between concentration and exposure status. For this part of the analysis, we omitted data collected from EE lines that were infected with *Spiroplasma* to eliminate any potential confounding effects. As the naïve flies were taken from the stock population and were not from a specific EE line, each poisoned vial of naïve flies was the same line ID.

Next, we used a generalised linear mixed effects model (GLMM) with binomial error to analyse the bioassay data from flies from the EE lines. This was done to determine the impact that sex ratio and the presence of *Spiroplasma* in the population had on adaptation to pesticide. Our maximal model had mortality as the response variable, and the fixed effects were log permethrin concentration, sex, sex ratio, *Spiroplasma* presence, an interaction between log concentration and sex, an interaction between log concentration and sex ratio and an interaction between log concentration and *Spiroplasma* presence. To account for non‐independence between repeated measures taken from EE lines at each concentration, we included EE line as a random factor with both random slopes and intercepts. Note that for this model, the term ‘*Spiroplasma* presence’ refers to the conditions under which certain individuals used to generate the dose–response data evolved, but is not directly indicative of the infection status of those individuals.

For the infected treatments, to monitor variation across EE lines in the number of infected families used to seed the next generation (i.e., to make sure certain infected EE lines were not seeded by a significanlty higher or lower number of families than other EE lines), we used a GLM with Poisson errors. Here, the number of infected families was used as the response variable and EE line was the sole fixed effect.

Finally, we analysed mortality across female‐only data from EE lines in which *Spiroplasma* was present in the population (treatment groups 3 and 4). This was done to determine whether there is an intrinsic impact of *Spiroplasma* infection on pesticide resistance. We used a GLMM where mortality was the response variable, and the fixed effects were: log concentration, and *Spiroplasma* infection status. EE line was included as a random factor with random slopes and intercepts. As stated earlier, infection status was ascertained by examining the sex ratio of the broods the individuals came from.

All of the analyses were run in R version 4.2.3 (R Core Team 2023). We inspected our dataset for anomalous values by graphically analysing line‐specific dose–response data. We also cross‐referenced spurious data with the experimental schedule to determine whether questionable data were temporally grouped and were therefore likely to be the result of laboratory error. Mixed effects models were analysed using the ‘lme4’ (Bates et al. 2015) and ‘glmmTMB’ (Brooks et al. 2017) packages. Model selection was conducted using likelihood ratio tests in which non‐significant terms were sequentially dropped from the model. Model fit was checked by analysing model residuals using the ‘DHARMa’ (Hartig 2022) package. We used the ‘ggeffects’ (Lüdecke 2018) package to generate 95% confidence intervals for our mixed effects model predictions.

## 3 Results

After plotting line‐specific dose–response data, we observed that mortality rates in EE lines 9–12 (which constituted a line from each treatment) were low relative to all other lines. Cross‐referencing data from these lines with our laboratory schedule showed that these data were all collected on the same day using the same pesticide stock dilutions. Thus, it is highly likely that these data are different from the results from other lines because of a laboratory error. We present analyses with these lines removed, but analyses of the full dataset generated results which were qualitatively the same (see supplementary material). Furthermore, our analysis showed that there was no variation in the number of infected families used to seed subsequent generations across infected EE lines (df = 7, *𝜒* ^2^ = 1.672, *p* = 0.976). We therefore assume that there was no confounding effect of genetic bottlenecking affecting evolution in our infected treatments.

### 3.1 Mortality in Selected Versus Naïve Flies

For the model comparing the EE lines with unexposed flies, model selection revealed that the best‐fitting model was the maximal model (df of residual deviance = 354) which included EE line, an interaction between pesticide concentration and sex, and an interaction between pesticide concentration and exposure status.

**FIGURE 2:**
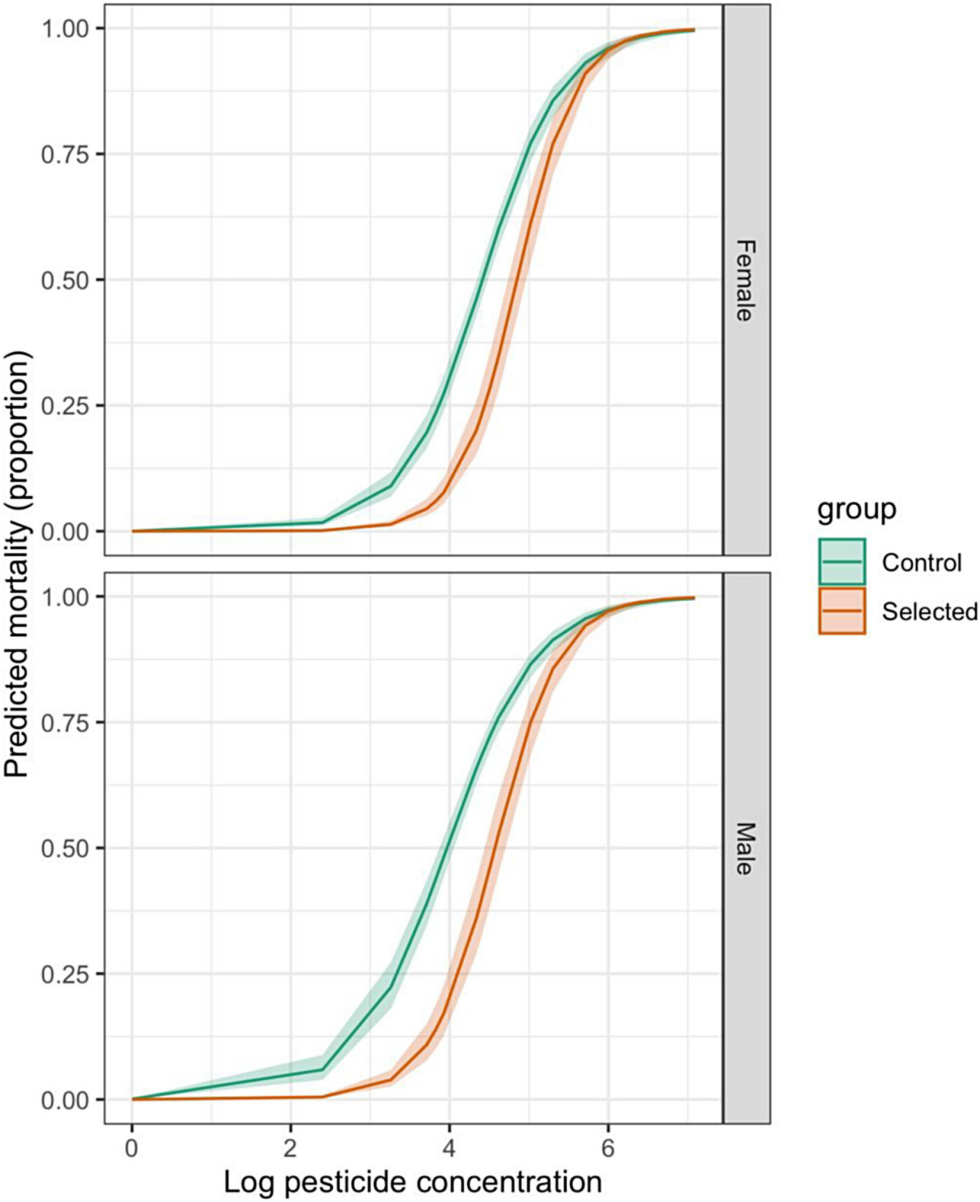
Sex‐specific differences in mortality in *Drosophila melanogaster* across a range of pesticide concentrations (μg/mL). The lines are predicted values from the fitted model with shaded areas indicating 95% confidence intervals. Green lines indicate mortality responses in naïve flies from populations that had not previously been exposed to pesticide, orange/red lines indicate mortality responses in flies from populations that had been exposed to pesticide for six generations prior.

The interaction between exposure status and log pesticide concentration was significant (*𝜒* ^2^ = 25.561, df = 1, *p* < 0.0001), with flies from EE lines demonstrating consistently lower mortality across the entire dosage range (Figure 2). There was also a significant interaction between sex and log pesticide concentration (*𝜒* ^2^ = 4.676, df = 1, *p* = 0.0306)—at intermediate concentrations males experienced higher mortality than females but at high pesticide concentrations male and female mortality were more similar (Figure 2).

The effect of EE line was significant (*𝜒* ^2^ = 199.211, df = 11, *p* < 0.0001); this suggests the presence of inter‐familial variation in pesticide resistance across our laboratory populations. Upon examining the main effect of sex, we saw that males had significantly higher mortality than females (*z* = 3.557, df = 1, *p* = 0.00038).

### 3.2 *Spiroplasma*, Sex Ratio Distortion and Mortality

**FIGURE 3:**
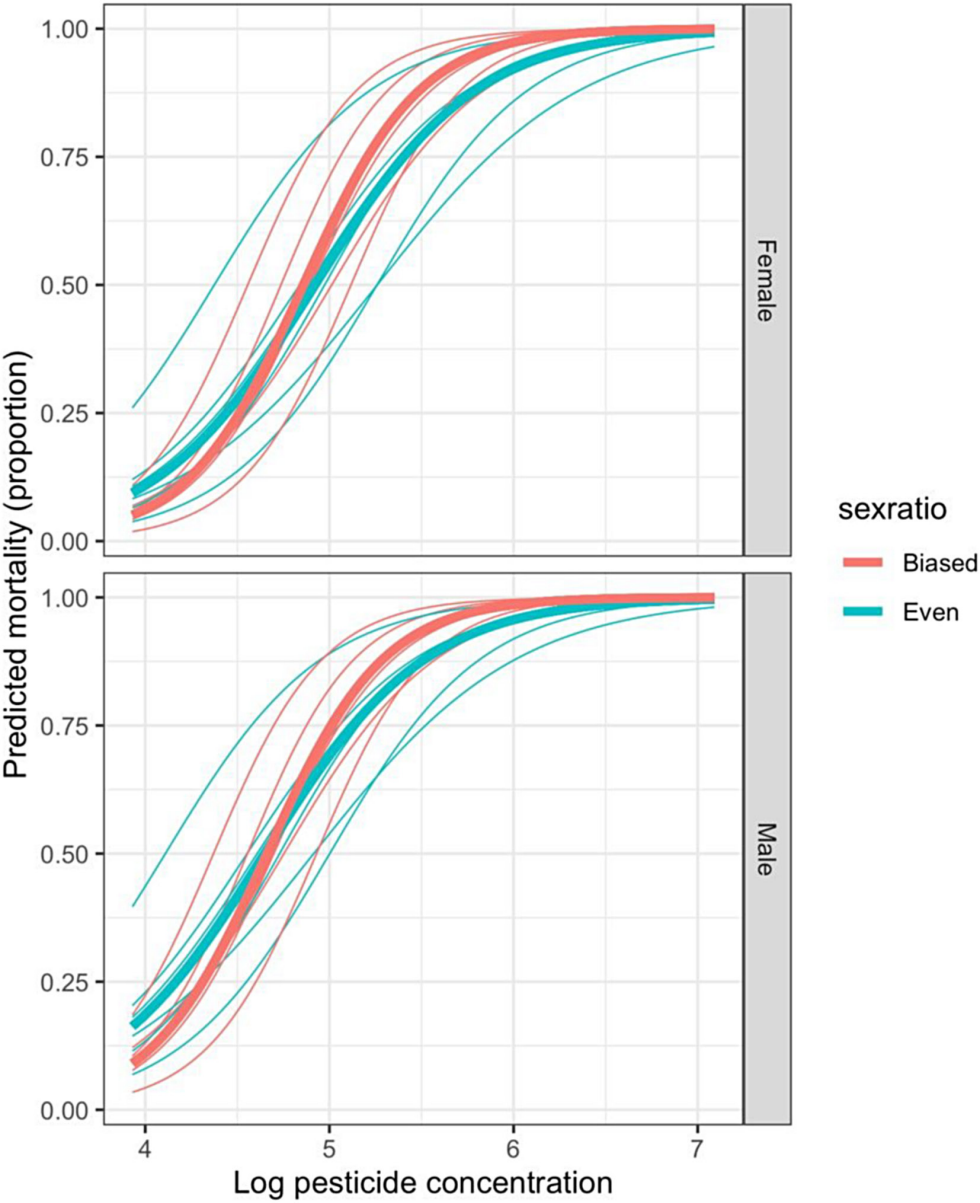
Sex‐specific differences in mortality in *Drosophila melanogaster* across a range of pesticide concentrations (μg/mL). Lines are predicted values from the fitted model with heavy lines indicating mean values and thin lines mortality for each of the experimental evolution lines. Orange/red lines indicate mortality responses from individuals that had evolved under six generations pesticide exposure in populations with a 3:1 female: male sex ratio. Blue lines indicate mortality responses from individuals that had evolved under six generations of pesticide exposure in populations with an even sex ratio.

For the model (df of residual deviance = 230) comparing dose–responses between the EE lines, the interaction between log concentration and sex ratio was retained in the final model (*𝜒* ^2^ = 7.344, df = 1, *p* = 0.00673) with flies from female‐biased treatments having a steeper mortality response as pesticide dose increased (Figure 3). The main effect of sex was also retained (*𝜒* ^2^ = 38.710, df = 1, *p* < 0.0001) with males having higher mortality than females in all EE lines. The interaction between *S*. *poulsonii* infection and dose was non‐significant, as was the main effect of infection (interaction: *𝜒* ^2^ = 0.753, df = 1, *p* = 0.386, main effect: *𝜒* ^2^ = 0.184, df = 1, *p* = 0.668), and the interaction between sex and dose was also not retained in the final model (*𝜒* ^2^ = 0.205, df = 1, *p* = 0.651).

Diagnostics for this model from DHARMa indicated a certain amount of underdispersion—as opposed to overdispersion this is rarely found but indicates that the variance of the residuals is less than would be expected (Gelman and Hill 2007; Zuur et al. 2009). Underdispersion can arise from overfitting of a model or if the model predicting some outlying data points rather better than might be expected (Zuur et al. 2009). We are confident that our model is not overfitted and we therefore follow the consensus as explained in Zuur et al. not to attempt correct for this, since if it has any effect, it will make the analysis more conservative.

Finally, upon analysing the female‐only data to compare mortality between females that were infected with *Spiroplasma* versus those that were not, model selection indicated that the presence of *Spiroplasma* had no explanatory power. Neither the interaction between log concentration and infection status, nor the main effect of infection status were significant (interaction: *𝜒* ^2^ = 0.883, df = 1, *p* = 0.347, main effect: *𝜒* ^2^ = 0.442, df = 1, *p* = 0.506). This suggests that *Spiroplasma* infections have no proximate effect on the resilience of hosts to pesticide.

## 4 Discussion

The use of sex ratio distortion to eradicate or hamper the proliferation of arthropod pests has been studied using theoretical models that focus on ecological impacts. Despite this, to the best of our knowledge, there has been no empirical test of the impact of sex ratio distortion on arthropod adaptation to pesticide. In this study, using an experimental approach with the fruit fly *Drosophila melanogaster*, we tested the impact of sex ratio distorting microbes (SRDMs) on arthropod adaptation to pesticide. First, we find that the skewed sex ratio that arises from the presence of sex ratio distorters did indeed change adaptation—flies that evolved under a female‐biased sex ratio demonstrated a different dose–response profile compared to flies that evolved under an even sex ratio. Specifically, at higher pesticide doses (>LD_50_), flies from skewed EE lines displayed higher mortality than those from even sex ratio EE lines (Figure 3), indicating a reduced adaptive response. Secondly, we found no evidence that the presence of *S. poulsonii* in the experimental lines had a direct impact on adaptation. Finally, we found an effect of sex across all of our experiments, with females being more resistant to pesticide than males (Figure 2).

We show that individuals from skewed EE lines experience a reduced adaptive response for pesticide doses of >LD_50_ than individuals from even sex ratio EE lines (Figure 3). Given that uneven sex ratios can create high levels of reproductive skew, which in turn limits gene flow (Chiba et al. 2023), it seems logical that reproductive skew was the mechanism driving the reduction in adaptation shown in our biased sex ratio treatments. However, as SRDMs create female‐biased sex ratios, this interpretation somewhat contradicts classic sexual selection theory, which, at least for species with female mate choice, would predict that a female bias would alleviate male–male competition for mates (Bateman 1948), thereby reducing reproductive skew. As such, the mechanistic explanation for our results is not obvious. One possibility is that the female‐biased treatments lead to a higher frequency of male mate choice (i.e., males being choosy with regard to the females they mate with), increasing reproductive skew among females. Indeed, plasticity in male choosiness in response to female availability has been thoroughly demonstrated in many species, including *D*. *melanogaster* (Byrne and Rice 2006; Edward and Chapman 2013; Rundus et al. 2015). Thus, SRDMs may lower adaptive potential via their impact on sexual selection; however, a study specifically measuring mate choice and adaptation in populations infected with SRDMs is required to formally test this prediction.

A prior theoretical study (Engelstädter and Hurst 2007) suggested that SRDM‐induced SR distortion can restrict adaptive potential by preventing the flow of alleles between the infected and uninfected portions of the host population. However, we found no evidence that the presence of SRDMs per se was responsible for any reduction in adaptation. The explanation for this discrepancy is most likely due to imperfect SRDM transmission rates, a condition that has been found repeatedly in laboratory studies involving SRDMs (Corbin et al. 2021; Ulrich et al. 2016). Imperfect transmission would allow adaptive alleles to flow between infected and uninfected portions of the population via the uninfected offspring of infected mothers dampening the adaptive effects of the gene flow restricting mechanism proposed by Engelstädter and Hurst (2007). In addition, we find no evidence of a direct impact of SRDMs on pesticide resistance (see Results); thus, we can conclude based on our data that sex ratio distortion alone was responsible for hindering adaptation. This result is consistent with a recent theoretical study which also found that sex ratio distortion was the dominant ecological mechanism driving increased extinction rates in populations infected with SRDMs (Fisher et al. 2024).

We also found that male *D*. *melanogaster* had a significantly higher mortality rate than females in response to pesticide exposure; this was true for both naïve flies (Figure 2) and flies from EE lines (Figure 3). Sex‐specific responses to environmental pressure are common in many species. Indeed, there are several relevant studies using *Drosophila* spp. that concur with our finding, and attribute this to sex‐specific selection of the *BA* allele, a pleiotropic allele which increases fecundity in females while also providing resistance to the common pesticide DDT (McCart, Buckling, and Ffrench‐Constant 2005). However, the fecundity effect of the *BA* allele for males has been shown to be either negative (Rostant et al. 2017; Smith et al. 2011) or neutral (Hawkes et al. 2016), and is thus not expected to be common in males from populations that are naïve to pesticide exposure. Nevertheless, it is also true that in the presence of a pesticide stressor, resistant alleles would be expected to increase in prevalence in the male populations via inheritance from resistant mothers. This may explain the disparity in adaptation we saw between males and females in our study (Figure 2).

While sex‐specific resistance to pesticides in arthropods has been shown previously, the impact of these sex‐specific differences on pest management is less well known. Based on our results, while inducing female‐biased sex ratios using SRDMs may restrict the rate of adaptation to pesticide, doing so will increase the frequency of the more resistant sex, potentially confounding the utility of SRDMs in a pest management scenario. Nevertheless, this will only be of concern to pest populations in which females are more resistant to pesticides than males, which is not the case for many non‐drosophilid pest species (de Lame et al. 2001; Rault et al. 2019). Our results highlight the importance of considering sex‐specific pesticide resistance when evaluating the use of sex ratio distorters for pest suppression.

This study provides the first empirical ‘proof of concept’ that SRDMs can reduce the evolutionary response of arthropods to pesticide stressors. We also highlight the possible confounding effects that intrinsic sex‐specific differences may have on a pest control strategy that relies on sex ratio distortion. Furthermore, the reproductive behaviour of specific pests will no doubt largely determine the extent to which sex ratio distortion will impact reproductive skew. To this end, we recommend that future work in this area focuses on long‐term semi‐natural experiments of real‐world pest species in order to generate more applied evidence of how SRDMs can be used to determine long‐term evolutionary responses to pesticides. An obvious candidate species for such a study would be *D. suzukii*, a widespread fruit pest which can be easily reared under semi‐natural conditions.

## Conflicts of Interest

The authors declare no conflicts of interest.

## Supporting information


Data S1.

Data S2.

## Data Availability

Raw data and analysis code are available as supporting information.
