# Supplementary material for: Do Sex Ratio Distorting Microbes Inhibit the Evolution of Pesticide Resistance? An Experimental Test
Source: Evol Appl. 2024 Oct 14;17(10):e70003. doi: 10.1111/eva.70003 (PMC11472798; doi:10.1111/eva.70003)
Supplement: Supplementary file 2 — Data S2. [file EVA-17-e70003-s001.pdf]

Male killer selection experiment analysis

AUTHOR
[redacted]

PUBLISHED
November 18, 2023

Setup

▼ Show the code

```r
library(ggplot2)
library(DHARMa)
```

```
This is DHARMa 0.4.6. For overview type '?DHARMa'. For recent changes, type news(packag
e = 'DHARMa')
```

▼ Show the code

```r
library(glmmTMB)
library(emmeans)
library(ggeffects)
```

```
Warning: package 'ggeffects' was built under R version 4.3.1
```

Load mortality data

▼ Show the code

```r
dose <- read.csv("DoseResponseData.csv")

dose$sexratio <-
  as.factor(ifelse(dose$Treatment == 1 |
                     dose$Treatment == 3, "Even", "Biased"))

dose$sexratio[dose$Stressed == "Control"] <- "Even"

dose$spiroplasma <-
  as.factor(ifelse(dose$Treatment >= 3, "Present", "Absent"))

dose$spiroplasma[dose$Stressed == "Control"] <- "Absent"

dose_sub1 <- subset(dose, Phenotype != "S")

dose_sub <- subset(dose, Phenotype != "S" & Stressed == "Y")

names(dose_sub)[3] <- "Sex"
names(dose_sub1)[3] <- "Sex"

dose_sub$Sex <- ifelse(dose_sub$Sex == "F", "Female", "Male")
dose_sub$Sex <- as.factor(dose_sub$Sex)
dose_sub1$Stressed <-
  ifelse(dose_sub1$Stressed == "Y", "Selected", "Control")
dose_sub1$Sex <- ifelse(dose_sub1$Sex == "F", "Female", "Male")
dose_sub1$Stressed <- as.factor(dose_sub1$Stressed)
dose_sub1$Sex <- as.factor(dose_sub1$Sex)
```

Visualise data

▶ Show the code

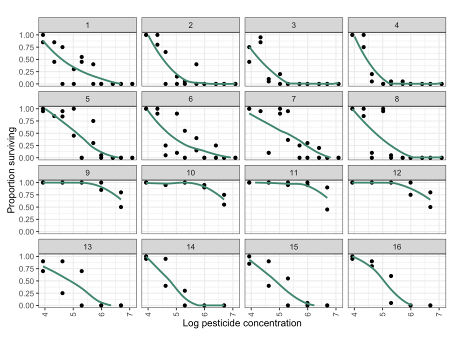

Proportion surviving plotted against log pesticide concentration for each line. Lines are a loess non-parametric smoother. NB dose 0 not shown because it makes everything messy.

Mostly fairly well behaved and low doses of pesticide are less lethal than high doses, which is reassuring. Lines 9-12 have much lower mortality than the others, however. We don't know if this is a real effect or if there was an error in, for example, making dilutions of pesticide. Given this uncertainty we will analyse the data with these lines removed, but note that the results are qualitatively the same wth these data included.

Have the selection lines evolved higher pesticide resistance?

We don't have control lines that weren't exposed to pesticide because this was not an important aim of the experiment and to do this would have been logistically impossible. We do have a lot of bioassay data from the stock population however and we can look at this and the selection lines. For the subsequent models where we are only analysing the differences between selection lines we will use random intercept and slope models to account for non-independence within lines. That isn't really possible here because we don't have lines for the stock bioassays but we will include line as a factor in a GLM to try to account for the non-independence.

```r
# remove problem lines identified above
testdat <- subset(dose_sub1, Line < 9 | Line > 12)

# remove data that was comparing infected and uninfected females
testdat <- subset(testdat, Line < 27)

# fit GLM. "stressed" indicates selection lines
testmod <-
  glm(
    cbind(deaths, survivors) ~ logConc * (Sex + Stressed) + as.factor(Line),
    family = binomial(link = "logit"),
    data = testdat
  )

# LRT to test significance of model terms
drop1(testmod, test = "Chisq")
```

```
Single term deletions

Model:
cbind(deaths, survivors) ~ logConc * (Sex + Stressed) + as.factor(Line)
                 Df Deviance    AIC     LRT  Pr(>Chi)
<none>                1819.7 2249.8
as.factor(Line)  11   2018.9 2427.0 199.211 < 2.2e-16 ***
logConc:Sex       1   1824.4 2252.4   4.676   0.03059 *
logConc:Stressed  1   1845.3 2273.3  25.561 4.287e-07 ***
---
Signif. codes:  0 '***' 0.001 '**' 0.01 '*' 0.05 '.' 0.1 ' ' 1
```

Significant dose by treatment interaction. Selection lines have substantially lower mortality at a given pesticide dose.

```r
# Visualise the selection effect

pred1 <-
  ggpredict(testmod, terms = c("logConc [all]", "Stressed", "Sex"))

p2 <-
  ggplot(data = pred1, aes(x = x, y = predicted, colour = group)) +
  geom_ribbon(aes(
    ymin = conf.low,
    ymax = conf.high,
    fill = group,
    colour = NA
  ),
  alpha = 0.3) +
  geom_line() + facet_grid(facet ~ .) +
  theme_bw() +
  scale_color_brewer(palette = "Dark2") + scale_fill_brewer(palette = "Dark2") +
  labs(x = "Log pesticide concentration", y = "Predicted mortality (proportion)")

p2
```

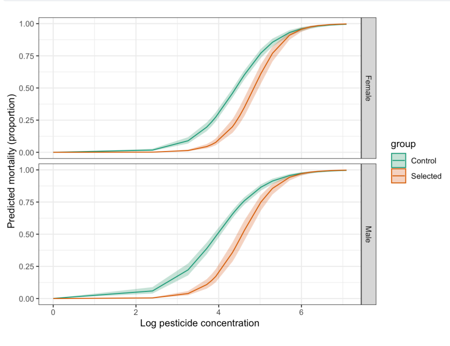

Modelling the effects of sex ratio and the presence of the male killer

```r
# Remove problem lines identified above
dose_sub2 <- subset(dose_sub, Line < 9 | Line > 12)

# Model with random slopes and intercepts.
# Using glmmTMB because of convergence problems with glmer
mod1a <-
  glmmTMB(
    cbind(deaths, survivors) ~ logConc * (Sex + sexratio + spiroplasma) +
      (logConc | Line),
    family = binomial(link = "logit"),
    data = dose_sub2
  )

# LRT test for interaction terms
drop1 (mod1a, test = "Chisq")
```

```
Single term deletions

Model:
cbind(deaths, survivors) ~ logConc * (Sex + sexratio + spiroplasma) +
    (logConc | Line)
                    Df    AIC    LRT Pr(>Chi)
<none>                 1484.3
logConc:Sex          1 1482.5 0.2110 0.646011
logConc:sexratio     1 1489.9 7.6155 0.005787 **
logConc:spiroplasma  1 1483.0 0.7527 0.385636
---
Signif. codes:  0 '***' 0.001 '**' 0.01 '*' 0.05 '.' 0.1 ' ' 1
```

```r
summary(mod1a)
```

```
 Family: binomial  ( logit )
Formula:
cbind(deaths, survivors) ~ logConc * (Sex + sexratio + spiroplasma) +
    (logConc | Line)
Data: dose_sub2

     AIC      BIC   logLik deviance df.resid
  1484.3   1522.5   -731.1   1462.3      227

Random effects:

Conditional model:
 Groups Name        Variance Std.Dev. Corr
 Line   (Intercept) 3.3131   1.8202
        logConc     0.1385   0.3722   -0.93
Number of obs: 238, groups:  Line, 12

Conditional model:
                           Estimate Std. Error z value Pr(>|z|)
(Intercept)                -14.7128     1.2814 -11.482  < 2e-16 ***
logConc                      3.0064     0.2613  11.506  < 2e-16 ***
SexMale                      0.2711     0.7766   0.349  0.72704
sexratioEven                 4.1196     1.3869   2.970  0.00298 **
spiroplasmaPresent          -0.9707     1.3814  -0.703  0.48225
logConc:SexMale              0.0721     0.1572   0.459  0.64643
logConc:sexratioEven        -0.8766     0.2838  -3.089  0.00201 **
logConc:spiroplasmaPresent   0.2445     0.2823   0.866  0.38658
---
Signif. codes:  0 '***' 0.001 '**' 0.01 '*' 0.05 '.' 0.1 ' ' 1
```

```r
# Remove non-significant terms
mod2a <- update(mod1a, ~ . - logConc:spiroplasma)

drop1(mod2a, test = "Chisq")
```

```
Single term deletions

Model:
cbind(deaths, survivors) ~ logConc + Sex + sexratio + spiroplasma +
    (logConc | Line) + logConc:Sex + logConc:sexratio
                 Df    AIC    LRT Pr(>Chi)
<none>              1483.0
spiroplasma       1 1481.2 0.1836 0.668276
logConc:Sex       1 1481.2 0.2032 0.652148
logConc:sexratio  1 1488.4 7.3412 0.006739 **
---
Signif. codes:  0 '***' 0.001 '**' 0.01 '*' 0.05 '.' 0.1 ' ' 1
```

```r
mod3a <- update(mod2a,  ~ . - spiroplasma)

drop1(mod3a, test = "Chisq")
```

```
Single term deletions

Model:
cbind(deaths, survivors) ~ logConc + Sex + sexratio + (logConc |
    Line) + logConc:Sex + logConc:sexratio
                 Df    AIC    LRT Pr(>Chi)
<none>              1481.2
logConc:Sex       1 1479.4 0.2045 0.651097
logConc:sexratio  1 1486.6 7.3494 0.006709 **
---
Signif. codes:  0 '***' 0.001 '**' 0.01 '*' 0.05 '.' 0.1 ' ' 1
```

```r
mod4a <- update(mod3a,  ~ . - logConc:Sex)

drop1(mod4a, test = "Chisq")
```

```
Single term deletions

Model:
cbind(deaths, survivors) ~ logConc + Sex + sexratio + (logConc |
    Line) + logConc:sexratio
                 Df    AIC    LRT  Pr(>Chi)
<none>              1479.4
Sex               1 1516.1 38.710 4.917e-10 ***
logConc:sexratio  1 1484.8  7.344  0.006729 **
---
Signif. codes:  0 '***' 0.001 '**' 0.01 '*' 0.05 '.' 0.1 ' ' 1
```

```r
# Final model
summary(mod4a)
```

```
 Family: binomial  ( logit )
Formula:
cbind(deaths, survivors) ~ logConc + Sex + sexratio + (logConc |
    Line) + logConc:sexratio
Data: dose_sub2

     AIC      BIC   logLik deviance df.resid
  1479.4   1507.2   -731.7   1463.4      230

Random effects:

Conditional model:
 Groups Name        Variance Std.Dev. Corr
 Line   (Intercept) 3.3637   1.8340
        logConc     0.1462   0.3824   -0.94
Number of obs: 238, groups:  Line, 12

Conditional model:
                     Estimate Std. Error z value Pr(>|z|)
(Intercept)          -15.3398     1.0683 -14.360  < 2e-16 ***
logConc                3.1571     0.2205  14.315  < 2e-16 ***
SexMale                0.6246     0.1015   6.154 7.57e-10 ***
sexratioEven           4.1059     1.3920   2.950  0.00318 **
logConc:sexratioEven  -0.8743     0.2881  -3.034  0.00241 **
---
Signif. codes:  0 '***' 0.001 '**' 0.01 '*' 0.05 '.' 0.1 ' ' 1
```

```r
#Removing lines 9:12 does not change the qualitative outcomes of the analysis
```

No effect of just having the male killer but a significant dose by sex ratio interaction.

Visualise the model predictions

▶ Show the code

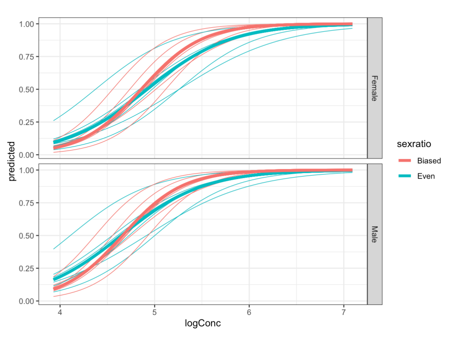

Predicted values for each line (narrow lines) and overall predicted values (heavy lines) for males and females from the fitted model. Lines with even sex ratios have lower mortality at high doses of pesticide.

Diagnostics for the fitted model

```r
# Check model fit using DHARMa residuals

simulationOutput <- simulateResiduals(fittedModel = mod4a, plot = F)

plot(simulationOutput)
```

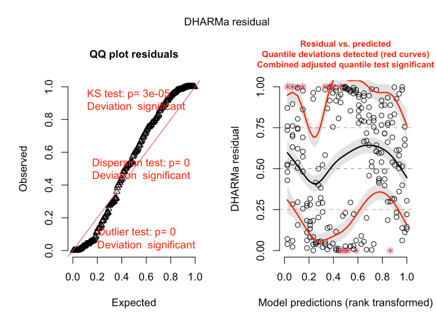


```r
testDispersion(simulationOutput)
```

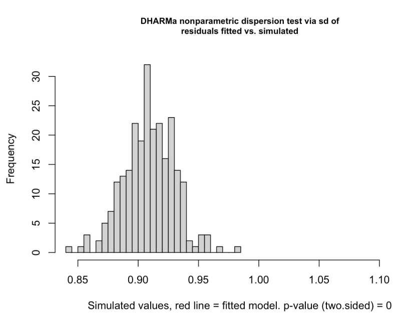


```
	DHARMa nonparametric dispersion test via sd of residuals fitted vs.
	simulated

data:  simulationOutput
dispersion = 1.2386, p-value < 2.2e-16
alternative hypothesis: two.sided
```

```r
testZeroInflation(simulationOutput)
```

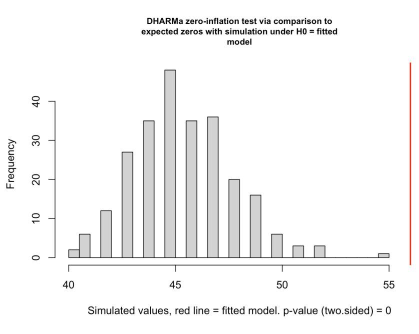


```
	DHARMa zero-inflation test via comparison to expected zeros with
	simulation under H0 = fitted model

data:  simulationOutput
ratioObsSim = 1.228, p-value < 2.2e-16
alternative hypothesis: two.sided
```

Some issues with the diagnostics which are mainly associated with underdispersion. This means that standard errors could be overestimated leading to type 2 errors. There's not a lot in the literature on dealing with underdispersion—overdispersion is more common and leads to type 1 errors so this has had a lot more attention. It's barely mentioned in most of the standard books. Crawley mentions it but no more, Gelman & Hill explain what it is but nothing more. Zuur et al. say to check if your model is overfitted and if it isn't then ignore it which I think is the approach I'll take. We don't have any important terms that close to significant and are being excluded from the model so it's unlikely that the increased type 2 error rate is causing any issues here so the best option is probably to ignore it.

Refitting with lines 9-12 included

```r
mod1 <-
  glmmTMB(
    cbind(survivors, deaths) ~ logConc * (sexratio + Sex + spiroplasma) +
      (logConc | Line),
    family = binomial(link = "logit"),
    data = dose_sub
  )


# Check significance with a likelihood ratio test

drop1(mod1, test = "Chisq")
```

```
Single term deletions

Model:
cbind(survivors, deaths) ~ logConc * (sexratio + Sex + spiroplasma) +
    (logConc | Line)
                    Df    AIC    LRT Pr(>Chi)
<none>                 1619.5
logConc:sexratio     1 1622.4 4.8831  0.02712 *
logConc:Sex          1 1620.1 2.5905  0.10750
logConc:spiroplasma  1 1618.4 0.8893  0.34566
---
Signif. codes:  0 '***' 0.001 '**' 0.01 '*' 0.05 '.' 0.1 ' ' 1
```

```r
mod2 <- update(mod1,  ~ . - logConc:spiroplasma)

drop1(mod2, test = "Chisq")
```

```
Single term deletions

Model:
cbind(survivors, deaths) ~ logConc + sexratio + Sex + spiroplasma +
    (logConc | Line) + logConc:sexratio + logConc:Sex
                 Df    AIC    LRT Pr(>Chi)
<none>              1618.4
spiroplasma       1 1616.5 0.0965  0.75601
logConc:sexratio  1 1621.0 4.6558  0.03095 *
logConc:Sex       1 1619.0 2.5744  0.10861
---
Signif. codes:  0 '***' 0.001 '**' 0.01 '*' 0.05 '.' 0.1 ' ' 1
```

```r
mod3 <- update(mod2,  ~ . - logConc:Sex)

drop1(mod3, test = "Chisq")
```

```
Single term deletions

Model:
cbind(survivors, deaths) ~ logConc + sexratio + Sex + spiroplasma +
    (logConc | Line) + logConc:sexratio
                 Df    AIC    LRT  Pr(>Chi)
<none>              1619.0
Sex               1 1667.3 50.293 1.325e-12 ***
spiroplasma       1 1617.0  0.086   0.76963
logConc:sexratio  1 1621.6  4.671   0.03067 *
---
Signif. codes:  0 '***' 0.001 '**' 0.01 '*' 0.05 '.' 0.1 ' ' 1
```

```r
mod4 <- update(mod3,  ~ . - spiroplasma)

drop1(mod4, test = "Chisq")
```

```
Single term deletions

Model:
cbind(survivors, deaths) ~ logConc + sexratio + Sex + (logConc |
    Line) + logConc:sexratio
                 Df    AIC    LRT  Pr(>Chi)
<none>              1617.0
Sex               1 1665.4 50.311 1.312e-12 ***
logConc:sexratio  1 1619.7  4.659   0.03089 *
---
Signif. codes:  0 '***' 0.001 '**' 0.01 '*' 0.05 '.' 0.1 ' ' 1
```

```r
summary(mod4)
```

```
 Family: binomial  ( logit )
Formula:
cbind(survivors, deaths) ~ logConc + sexratio + Sex + (logConc |
    Line) + logConc:sexratio
Data: dose_sub

     AIC      BIC   logLik deviance df.resid
  1617.1   1646.3   -800.5   1601.1      278

Random effects:

Conditional model:
 Groups Name        Variance Std.Dev. Corr
 Line   (Intercept) 5.1465   2.2686
        logConc     0.2021   0.4495   -0.47
Number of obs: 286, groups:  Line, 16

Conditional model:
                     Estimate Std. Error z value Pr(>|z|)
(Intercept)          15.66372    1.13450  13.807  < 2e-16 ***
logConc              -2.94650    0.21626 -13.625  < 2e-16 ***
sexratioEven         -3.19598    1.49026  -2.145   0.0320 *
SexMale              -0.67251    0.09603  -7.003  2.5e-12 ***
logConc:sexratioEven  0.66888    0.28703   2.330   0.0198 *
---
Signif. codes:  0 '***' 0.001 '**' 0.01 '*' 0.05 '.' 0.1 ' ' 1
```

Minimal model is the same as the one fitted to the dataset with lines 9-12 removed, although the confidence is reduced in the model fitted to the full data.
